# Oxygen Plasma Functionalization of Activated Carbon Pellets for Hazardous HCl Gas Mitigation: Balancing Surface Oxygenation and Pore Preservation

**DOI:** 10.3390/toxics14060459

**Published:** 2026-05-24

**Authors:** Min Seong Han, Jong Hyun Lee, Do Hyun Kim, Byong Chol Bai

**Affiliations:** Department of Chemical Engineering, Daejin University, Pocheon 11159, Republic of Korea

**Keywords:** activated carbon, HCl adsorption, oxygen plasma treatment, pitch, pellet

## Abstract

Hydrogen chloride (HCl) is a hazardous acidic gas released from industrial processes and waste-treatment systems, posing risks to human health, process safety, and the surrounding environment. Accordingly, there is a need for practical adsorbent materials that can reduce HCl exposure without generating secondary liquid waste. In this study, pitch-based activated carbon pellets were surface-functionalized by oxygen plasma treatment to improve fixed-bed HCl removal performance. Plasma treatment was applied for 1, 2, and 4 min, and the resulting changes in surface chemistry, pore structure, and adsorption behavior were investigated using SEM, XPS, N_2_ adsorption–desorption analysis, and breakthrough experiments. Oxygen plasma treatment increased the oxygen-containing surface functionalities of the pellets while largely preserving pellet morphology. Under moderate treatment conditions (1–2 min), the BET surface area and pore volume were mostly maintained, whereas prolonged treatment (4 min) reduced the accessible pore structure. In fixed-bed adsorption tests, the sample treated for 1 min showed the longest breakthrough behavior and the highest HCl uptake among the tested samples, while the sample treated for 2 min exhibited the shortest mass transfer zone and the highest bed utilization. These results indicate that controlled oxygen plasma treatment can improve the removal of hazardous HCl gas by balancing surface functionalization and pore preservation. The findings suggest that plasma-functionalized activated carbon pellets are a promising option for toxic acidic gas mitigation in air pollution control and waste-treatment applications.

## 1. Introduction

Hydrogen chloride (HCl) is widely used in semiconductor manufacturing and electronic material processes [1]. It is also released during the incineration of plastics, polyvinyl chloride (PVC), and other organic wastes, and is one of the representative acidic by-products in such systems [2]. HCl is highly corrosive and can damage metal piping and process equipment, and it may also reduce process stability through catalyst poisoning [3]. In air, it readily reacts with moisture and can form acid fog or acid rain, causing environmental problems such as soil and water acidification. Exposure to HCl can also irritate the eyes, skin, and respiratory tract, even at relatively low concentrations [4]. Several methods have been used to remove acidic gases, including catalytic decomposition, wet scrubbing, chemical neutralization, and adsorption. However, each method has drawbacks. Catalytic systems can suffer from deactivation, while wet scrubbing and neutralization generate wastewater and may cause secondary contamination [5,6,7]. For this reason, adsorption has been considered a practical option, especially for low-concentration gas treatment, because the process is simple and does not require additional liquid-phase treatment step [8].

Gas adsorption is generally classified into physical adsorption and chemical adsorption. Physical adsorption is mainly governed by weak interactions such as van der Waals forces or electrostatic attraction, and its reversibility is an advantage. However, the interaction strength is limited. Chemical adsorption provides stronger interaction and higher selectivity, but it depends strongly on surface chemistry and is often disadvantageous for regeneration [9,10,11]. Zeolites, activated carbons, and metal oxides have all been studied for acidic gas removal [12,13,14]. Among them, activated carbon is still one of the most commonly used adsorbents, because it has a well-developed micropore structure and can remove hazardous gases without generating wastewater or serious secondary pollution [15,16]. The limitation of activated carbon is its surface property. The carbon surface is basically nonpolar; so, its interaction with polar acidic gases is not strong. This is an important issue for HCl, because HCl has a strong dipole moment. In untreated activated carbon, pore adsorption alone is often not enough to achieve high HCl removal efficiency, particularly at low concentrations [17,18,19].

For this reason, many studies have tried to modify the carbon surface by introducing heteroatom-containing functional groups such as oxygen, nitrogen, and sulfur [20]. Surface modification methods are generally divided into wet and dry processes [21]. Wet treatments, such as acid/base oxidation and chemical doping, are effective for introducing functional groups, but they require chemical reagents and additional washing or post-treatment steps [22,23,24,25]. Thermal oxidation can also introduce oxygen-containing functional groups onto carbon surfaces, but high-temperature treatment may cause carbon burn-off, pore widening, or partial damage to the porous framework. In contrast, dry treatments, including plasma and sputtering, are simpler in this respect. Oxygen plasma treatment can be carried out quickly under mild conditions without generating liquid waste, and it enables surface selective modification of carbon materials. This is advantageous for pelletized activated carbon because surface functionalization should be achieved while preserving the accessible pore structure [26,27,28]. Among these methods, oxygen plasma is useful for introducing oxygen-containing functional groups onto the carbon surface [29]. These groups change the surface electronic state and increase the surface polarity. Such a change can improve the interaction with polar gas molecules such as HCl. Previous studies have suggested that oxygen-containing groups can affect adsorption through dipole–dipole interaction or hydrogen bonding [30,31,32,33]. Recent studies have also emphasized that detailed surface characterization, including FTIR and XPS analyses, is essential for correlating oxygen-containing functional groups and surface property changes with adsorption behavior in carbon-based materials [34]. However, industrial HCl treatment still relies mainly on wet scrubbing and neutralization, since those methods are already established in practice. In addition, many previous plasma studies have focused more on nitrogen plasma and on increasing surface basicity to promote chemical adsorption [35,36,37,38]. Studies dealing with oxygen plasma for HCl adsorption are relatively limited. The combined effect of oxygen functionalization and pore structure change has also not been discussed clearly.

In this study, pitch-based activated carbon pellets were treated with oxygen plasma, and the changes in surface chemistry, pore structure, and HCl adsorption behavior were examined. The objective of this study was to determine whether oxygen plasma treatment can improve HCl adsorption while minimizing damage to the pore structure of pelletized activated carbon.

## 2. Materials and Methods

### 2.1. Materials

Petroleum-based pitch (softening point: 250 °C; Smart Korea Co., Daejeon, Republic of Korea) was used as the carbon precursor, and potassium hydroxide (KOH, 95.0%; Samchun Pure Chemical Co. Ltd., Pyeongtaek, Republic of Korea) was employed as the chemical activating agent. For pelletization, distilled water, an acrylic binder (Unisol Chemicals Co., Ansan, Republic of Korea), and carboxymethyl cellulose (CMC, Unisol Chemicals Co., Ansan, Republic of Korea) were used. Plasma treatment was conducted using high-purity oxygen gas (O_2_, 99.999%, Samjung Gas Co., Paju, Republic of Korea). For the HCl adsorption experiments, nitrogen gas (N_2_, 99.999%, Samjung Gas Co., Paju, Republic of Korea) was used as the carrier gas, and a standard HCl gas mixture (50 mg/L in N_2_, Rigas Co., Daejeon, Republic of Korea) was used as the feed gas.

### 2.2. Preparation of Activated Carbons

Petroleum pitch was finely ground using a hand mixer. The pitch was stabilized in air by heating to 260 °C at a rate of 1 °C/min in a stabilization furnace and holding at 260 °C for 1 h. The stabilized pitch was then mixed with KOH at a weight ratio of 1:4. The mixture was transferred to a tube furnace and purged with N_2_ at 100 cm^3^/min under standard conditions for 1 h 30 min before activation. The temperature was raised to 850 °C at 10 °C/min and maintained for 1 h for chemical activation. After activation, residual potassium species such as unreacted KOH, K_2_CO_3_, and metallic K remained in the sample. The activated product was washed thoroughly with distilled water until the pH dropped below 8 and was then dried at 80 °C for 24 h.

### 2.3. Pelletization and Oxygen Plasma Treatment

Activated carbon, distilled water, acrylic binder, and CMC were mixed at a weight ratio of 1:1:0.14:0.01. The mixture was kneaded to obtain a homogeneous paste and then extruded using a pelletizer (Blue Science Co., Daejeon, Republic of Korea) at 25 rpm. The resulting pellets were cylindrical, with a diameter of 3 mm and a length of 5–8 mm. The pellets were dried at room temperature for 24 h and then dried again at 80 °C for 24 h. Oxygen plasma treatment was carried out using a vacuum plasma system (Femto Science Co., Yongin, Republic of Korea). After the chamber was evacuated, oxygen gas was introduced at 100 cm^3^/min under standard conditions. The plasma power was fixed at 500 W, and the frequency was 50 kHz. Since plasma treatment mainly affects the exposed surface, both sides of the pellets were treated separately. Each side was treated for 1, 2, or 4 min. The untreated sample was denoted as PR, and the plasma-treated samples were denoted as P1, P2, and P4 according to the treatment time.

### 2.4. HCl Adsorption Experiment

Before sample loading, the glass column and gas lines were purged with N_2_. The samples (PR, P1, P2, and P4) were randomly packed into a glass column with an inner diameter of 1.6 cm to form a fixed bed. The pellets were not intentionally aligned along the gas flow direction. For each run, 1.00 g of sample was used, and the bed height was fixed at 2.5 cm at room temperature. A standard HCl gas mixture with an inlet concentration of 50 mg/L was introduced at a total flow rate of 1000 cm^3^/min under standard conditions. The outlet HCl concentration was monitored every second using an IR-based HCl gas analyzer. The adsorption test was continued until the outlet concentration approached the inlet concentration. After the adsorption experiment, N_2_ was flowed through the column to remove the remaining HCl gas.

### 2.5. Characterization of the Samples

Surface morphology at each processing stage, including the SP250 pitch, stabilized pitch, pelletized activated carbon and plasma-treated samples, was observed using scanning electron microscopy (SEM; VEGA3, Tescan, Kohoutovice, Czech Republic). The morphological changes after stabilization, KOH activation, and oxygen plasma treatment were compared. The surface chemical composition was analyzed by X-ray photoelectron spectroscopy (XPS; Nexsa, Thermo Fisher Scientific, Waltham, MA, USA). High-resolution C1s and O1s spectra were deconvoluted to examine the oxygen-containing functional groups introduced by plasma treatment and the resulting changes in surface elemental composition. The textural properties of the samples were analyzed by N_2_ adsorption–desorption isotherms at 77 K, and the specific surface area was calculated using the Brunauer–Emmett–Teller (BET) method (Tristar 3020, Micromeritics, Norcross, GA, USA). The HCl adsorption performance was evaluated from breakthrough curves obtained by continuously monitoring the outlet HCl concentration using a gas detector (FIX800, Wandi Co., Gunpo, Republic of Korea). The adsorption capacity was calculated from the breakthrough curve using the inlet concentration and total flow rate, and the adsorption performances of PR, P1, P2, and P4 were compared.

## 3. Results and Discussion

### 3.1. SEM Analysis of Surface Morphology

#### 3.1.1. Surface Morphology Evolution During Stabilization and KOH Activation

**Figure 1:**
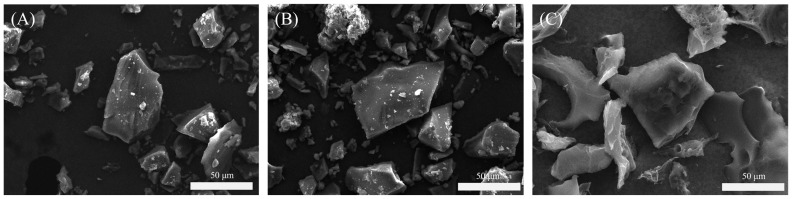
SEM images of samples at different stages of activated carbon production: (**A**) SP250 pitch, (**B**) stabilized pitch, and (**C**) KOH-activated sample.

SEM images are shown in Figure 1 to enable examination of the structural changes during stabilization and KOH activation. Figure 1A shows the SP250 pitch before stabilization. As shown in Figure 1B, the stabilized pitch still exhibited a relatively dense and smooth surface without a noticeable structural change. In contrast, Figure 1C shows that the morphology changed clearly after KOH activation. The activated sample exhibited fractured surfaces, and the exposed surfaces became smoother, which appears to be associated with etching of the pitch-based carbon matrix. This morphology is generally attributed to the reaction between KOH-derived potassium species and the pitch-based carbon matrix during activation, which causes etching of the carbon framework and leaves smoother exposed surfaces after structural collapse [38,39,40].

#### 3.1.2. Surface Morphology After Oxygen Plasma Treatment

**Figure 2:**
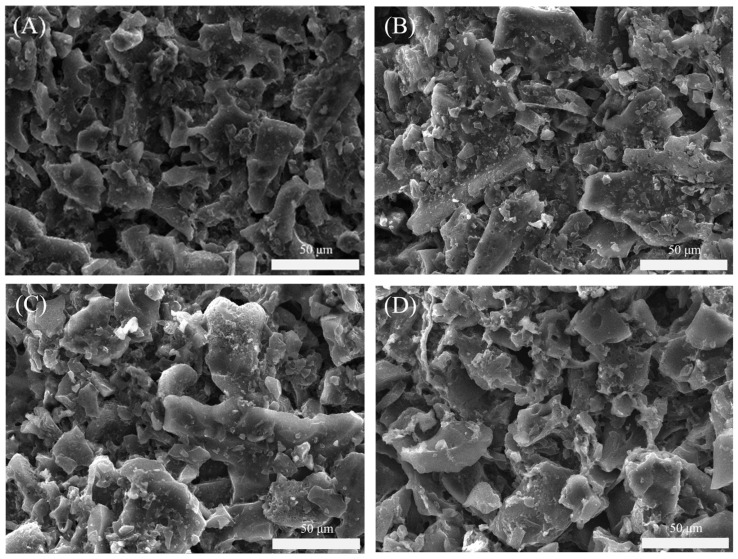
SEM images of pelletized activated carbon samples according to oxygen plasma treatment: (**A**) PR, (**B**) P1, (**C**) P2, and (**D**) P4.

SEM images of PR, P1, P2, and P4 are shown in Figure 2. In all samples, the overall morphology of the pelletized activated carbon was retained after oxygen plasma treatment. Surface-adhered phases were observed on the carbon particles in both PR and the plasma-treated samples, and no obvious detachment or severe structural collapse was observed after plasma treatment. Compared with PR, P1, and P2 did not show a clear morphological difference at this magnification. In contrast, P4 still retained the overall morphology, but locally more irregular surface features and edge erosion were observed, suggesting that prolonged plasma treatment may begin to etch the exposed carbon surface. Overall, these results indicate that oxygen plasma treatment mainly affected the surface chemistry rather than the visible morphology of the pellets, although excessive treatment could induce limited surface etching [26,27,28,29,41].

### 3.2. Surface Elemental Composition and Chemical States

**Figure 3:**
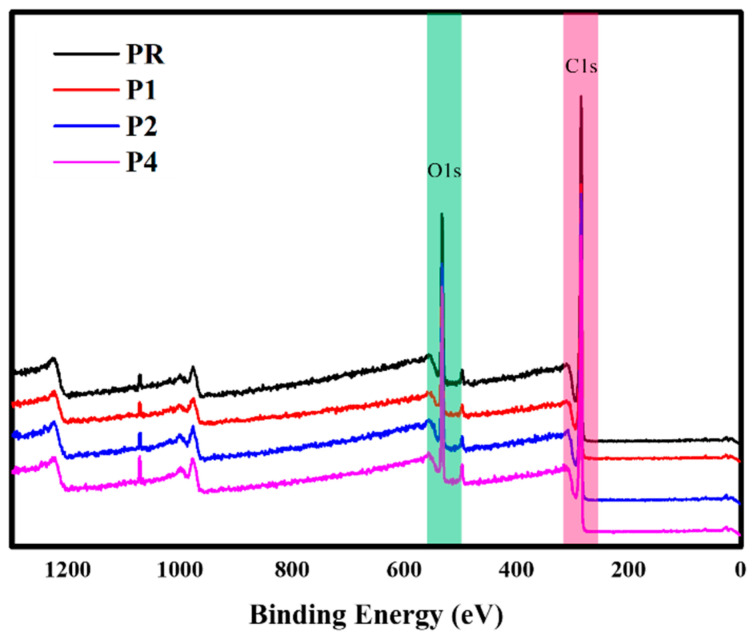
XPS survey spectra of samples with different plasma treatments.

**Figure 4:**
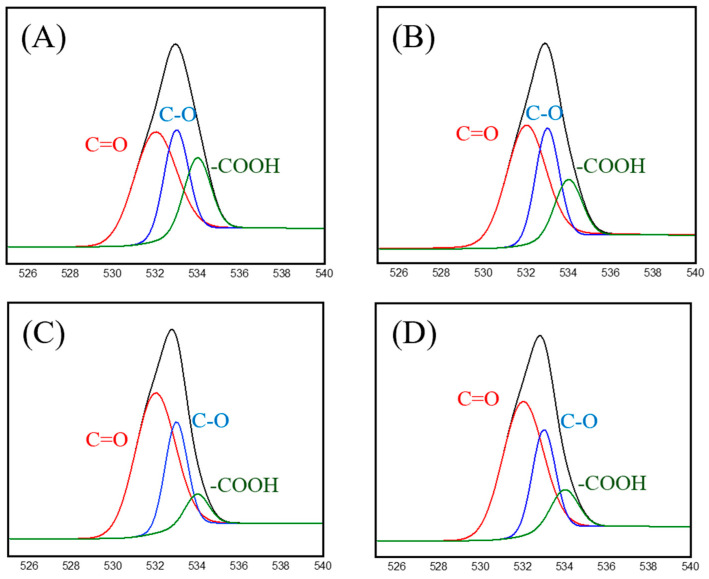
Deconvoluted O1s XPS spectra of samples with different plasma treatments: (**A**) PR, (**B**) P1, (**C**) P2, and (**D**) P4.

As shown in Figure 3, the XPS survey spectra exhibited two major peaks in the binding energy ranges of approximately 284–290 eV and 531–534 eV, which were assigned to C1s and O1s, respectively [42]. The O1s peak intensity gradually increased from PR to P4 with increasing plasma treatment time, indicating an increase in oxygen-containing surface functional groups. To examine the evolution of these oxygen species in more detail, the O1s spectra were deconvoluted into their constituent components, as presented in Figure 4. The deconvoluted O1s spectra consisted of three main components associated with carbonyl groups (C=O, ~530.9–532.0 eV), hydroxyl/ether groups (C-O, ~532.0–533.0 eV), and carboxyl groups (-COOH, ~533.0–534.5 eV) [43,44]. As shown in Figure 4, the PR sample exhibited relatively low contributions from the C-O and C=O components, whereas the plasma-treated samples showed progressively increased O1s intensities and enlarged relative peak areas corresponding to hydroxyl, ether, and carboxyl groups. These findings indicate that oxygen plasma treatment successfully enriched the activated carbon surface with polar oxygen-containing functional groups [45]. The surface elemental compositions obtained from XPS analysis are summarized in Table 1.

The introduction of these groups may improve the affinity of the adsorbent toward polar acidic gas molecules such as HCl through dipole–dipole interactions and hydrogen bonding [46]. Hydroxyl/ether, carbonyl, and carboxyl groups may increase the surface polarity of activated carbon and provide possible interaction sites for HCl molecules [46,47]. However, the HCl adsorption performance did not increase monotonically with the O1s content. This suggests that the adsorption performance was not controlled only by the total amount of oxygen-containing functional groups. The accessibility of these functional groups within the pore structure is also important. Therefore, the higher adsorption capacity of P1, despite its lower O1s intensity than P2 and P4, can be explained by the balance between increased surface polarity and preservation of accessible pore structure [47].

### 3.3. BET Surface Area and Pore Structure Analysis

**Figure 5:**
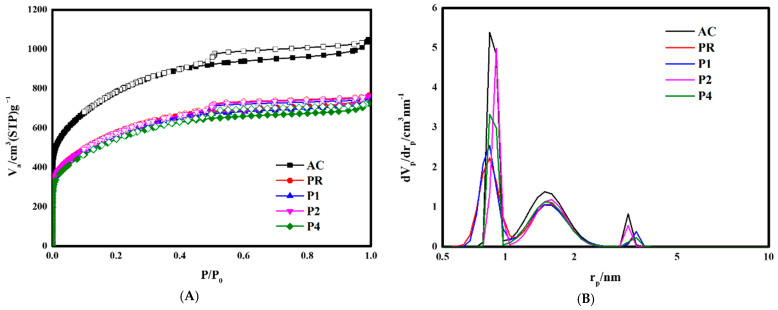
Textural properties of samples with different plasma treatments: (**A**) N_2_ adsorption–desorption isotherms and (**B**) pore size distribution curves.

The textural properties of the samples were characterized by N_2_ adsorption–desorption isotherms, and the results are presented in Figure 5 and Table 2. The BET surface area of activated carbon before pelletization (AC) was 2791.30 m^2^/g, whereas that of PR decreased to 2053.60 m^2^/g. This reduction was attributed to the blockage of activated carbon pores by the acrylic binder and CMC introduced during pelletization [48].

Following oxygen plasma treatment, the BET surface areas of PR, P1, and P2 were 2053.60, 2019.00, and 2039.60 m^2^/g, respectively, indicating no substantial change. Similarly, the total pore volumes of PR, P1, and P2 were 1.17, 1.16, and 1.17 cm^3^/g, respectively, suggesting that oxygen plasma treatment up to P2 did not significantly alter the pore structure of the activated carbon. In contrast, for P4, the BET surface area decreased to 1928.00 m^2^/g, and the total pore volume decreased to 1.12 cm^3^/g. This suggests that excessive plasma treatment can lead to the loss of accessible pore volume or to structural damage to the carbon framework, thereby lowering the adsorption capacity [49,50].

As shown in Figure 5B, the pore size distribution was concentrated mainly below 2 nm, indicating that the samples were predominantly microporous. In addition, the micropore fraction increased from 76.92% for PR to 86.21%, 84.62%, and 87.50% for P1, P2, and P4, respectively. The increase in micropore fraction after plasma treatment suggests that the relative contribution of micropores became larger than that of wider pores. This may be related to slight changes in the outer pore region or removal of the surface-adhered phases under moderate plasma treatment. In contrast, prolonged treatment appears to reduce the overall accessible pore volume, as observed for P4.

### 3.4. HCl Adsorption Performance

Breakthrough curves (*C_t_*/*C*_0_ vs. time) were drawn to analyze the adsorption of HCl in the upflow fixed-bed column; the data were evaluated using the following equations (Effluent volume: *V_eff_*) [50]:

(1) $$V_{eff}=Qt_{total}$$

where *t_total_* represents the total operation time (min), and *Q* represents the flow rate (mL/min). The total uptake capacity (*q_total_*) is given by Equation (2) [51]:

(2) $$q_{total}=\frac{Q}{1000}\int_{0}^{t_{total}}C_{ad}dt$$

where *C_ad_* is the adsorbed HCl concentration, defined as the difference between the inlet and outlet HCl concentrations (*C_ad_* = *C_0_* − *C_t_*). The total amount of HCl flowing into the column (*m_total_*, mg) is defined as follows [52]:

(3) $$m_{total}=\frac{C_{0}V_{eff}}{1000}$$

The total HCl removal (*R_total_*, %) was calculated as follows [52]:

(4) $$R_{total}=\frac{q_{total}}{m_{total}}\times 100$$

The mass transfer zone (*MTZ*), *Z_m_*, is defined as follows [53]:

(5) $$Z_{m}=Z\left(1-\frac{t_{0.05}}{t_{0.95}}\right)$$

where *Z_m_* is the length of the *MTZ* (cm), *Z* is the bed height (cm), *t*_0.05_ is the time at the breakthrough (min), and *t*_0.95_ is the time at saturation (min). The column usage of the fixed-bed column adsorption device (*ƒ*) is defined as follows [53]:

(6) $$f=\left(1-\frac{0.5\times MTZ}{bed\;height}\right)\times 100$$

**Figure 6:**
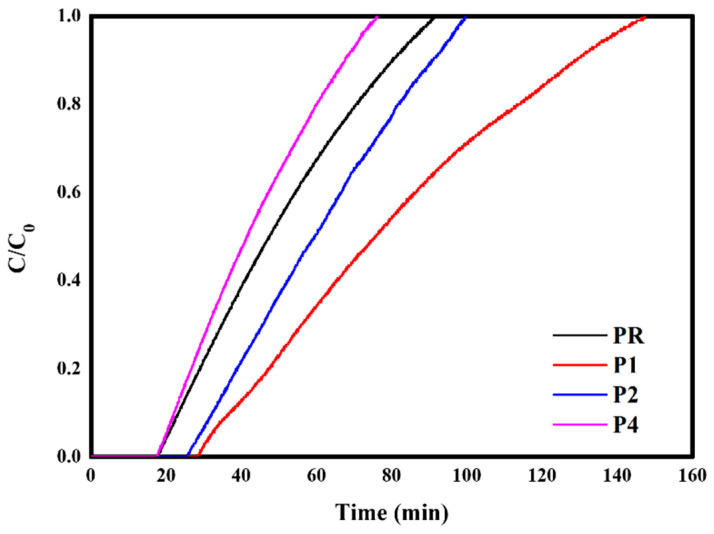
Breakthrough curves for HCl adsorption on PR, P1, P2, and P4 under identical fixed-bed conditions.

Figure 6 presents the breakthrough curves obtained from the fixed-bed HCl adsorption experiments for PR, P1, P2, and P4. All experiments were carried out under identical operating conditions (*Q* = 1000 mL/min, *C*_0_ = 50 mg/L, *Z* = 2.5 cm), allowing direct comparison of the adsorption behavior of each sample.

As summarized in Table 3, P1 showed the highest adsorption capacity and the longest breakthrough and saturation times among the tested samples. The *t_total_* of P1 reached 147.4 min, which was markedly longer than that of PR (91.3 min), P2 (99.7 min), and P4 (76.0 min). In addition, P1 showed the longest breakthrough time (*t*_0.05_ = 31.6 min) and saturation time (*t*_0.95_ = 137.1 min), indicating that HCl breakthrough was delayed most effectively in this sample. The adsorption capacity of P1 was also the highest at 995.26 mg/g. These results indicate that oxygen plasma treatment for 1 min was the most effective condition for improving the HCl adsorption of pelletized activated carbon.

The improved HCl adsorption performance of P1 is attributed to the introduction of oxygen-containing functional groups while preserving the pore structure. BET analysis showed no significant change in surface area or pore volume for PR, P1, and P2, whereas P4 exhibited reduced textural properties after excessive plasma treatment. In addition, XPS results confirmed an increase in O1s intensity after plasma treatment, indicating successful introduction of oxygen-containing functional groups onto the carbon surface. These polar oxygen-containing groups may improve the interaction between the carbon surface and HCl through hydrogen bonding, dipole–dipole interactions, and possible acid–base interactions. However, excessive surface oxidation may also induce pore blockage or partial collapse of micropores, thereby reducing the accessible surface area and lowering the adsorption capacity [54,55].

Although P1 showed the highest adsorption capacity, P2 showed the shortest mass transfer zone (*L_MTZ_* = 1.75 cm) and the highest bed utilization (65.03%), indicating more efficient use of the packed bed. In contrast, P1 showed a broader mass transfer zone (*L_MTZ_* = 1.92 cm) and a lower bed utilization (61.52%), suggesting that the adsorption front was distributed over a wider region of the bed. This difference suggests that a higher adsorption capacity did not necessarily correspond to better bed utilization. In P2, the adsorption front moved through the bed more effectively, even though the total uptake was lower than that of P1.

Within the tested conditions, oxygen plasma treatment for 1 and 2 min improved the HCl adsorption behavior of pelletized activated carbon. In particular, the 1 min treatment showed the highest adsorption capacity and the longest breakthrough behavior, whereas the 2 min treatment showed the most efficient bed utilization. The 4 min treatment reduced the adsorption performance, suggesting that excessive plasma exposure is unfavorable. Further multiobjective optimization using additional treatment times, such as below 1 min or between 1 and 2 min, will be considered in future work.

Based on the present results, a possible explanation for the HCl adsorption behavior of the plasma-treated samples is as follows. In P1, oxygen plasma treatment introduced oxygen-containing functional groups without a significant loss of surface area or pore volume. Under this condition, the polar surface groups appear to improve the affinity of the carbon surface toward HCl, while the microporous structure remains largely accessible for adsorption. This is considered to be the main reason why P1 showed the highest breakthrough time and adsorption capacity. In P2, the amount of oxygen-containing functional groups increased further, but the overall adsorption capacity was lower than that of P1. This suggests that additional surface oxidation did not directly lead to higher uptake, although it may have contributed to improved bed utilization and a shorter mass transfer zone. In P4, prolonged plasma treatment increased the surface oxygen content further, but the BET surface area and pore volume decreased. This indicates that excessive plasma treatment can reduce the accessibility of adsorption sites despite the increase in oxygen-containing groups. Therefore, the HCl adsorption behavior in this system appears to be governed by a balance between surface functionalization and preservation of the pore structure [47,55,56,57].

It should also be noted that this study has some limitations. The HCl adsorption tests were carried out at a single inlet concentration, flow rate, and temperature. Therefore, the present results should be understood as a comparative evaluation of plasma treatment time under fixed experimental conditions, rather than as a complete optimization of the adsorption process. Fixed-bed kinetic models such as the Thomas, Yoon–Nelson, and Bohart–Adams models were not applied in this study because additional data obtained under different operating conditions would be required for more reliable model interpretation. In addition, regeneration tests were not performed in this study. Since desorption and re-adsorption may change both the oxygen-containing surface groups and the accessible pore structure, future work should examine regeneration behavior together with post-regeneration XPS and pore-structure analysis.

## 4. Conclusions

In this study, pitch-based activated carbon pellets were treated with oxygen plasma to improve HCl adsorption performance, and the effects of treatment time on surface functional groups, pore structure, and fixed-bed adsorption behavior were evaluated. Oxygen plasma treatment introduced oxygen-containing functional groups onto the carbon surface without causing noticeable morphological damage to the pellet structure. Under 1 and 2 min treatment conditions, the pore structure was largely preserved, whereas excessive plasma exposure led to reductions in the BET surface area and pore volume.

Among the samples, P1 showed the highest adsorption capacity and the longest saturation time. These results indicate that 1 min oxygen plasma treatment enhanced the affinity of pelletized activated carbon toward HCl while maintaining its porous structure. In contrast, P2 exhibited the shortest mass transfer zone and the highest bed utilization, indicating more efficient use of the packed bed. This means that the optimal treatment condition can differ depending on which performance factor is given priority. P4 exhibited a reduced surface area, lower pore volume, and decreased adsorption capacity, indicating that excessive plasma exposure limited the accessibility of adsorption sites despite the increased surface oxygen functionality.

Overall, controlled oxygen plasma treatment was effective for improving HCl adsorption by pelletized activated carbon. In particular, the 1 min treatment provided the highest adsorption capacity, whereas the 2 min treatment resulted in the most efficient bed utilization. These results show that both surface functionalization and pore structure preservation should be considered in the design of activated carbon adsorbents for acidic gas removal.

## Figures and Tables

**Table 1:** Surface elemental composition of samples with different plasma treatments obtained from XPS analysis.

XPS Peak	Sample			
	PR	P1	P2	P4
C1s (at%)	83.06	82.65	80.95	80.33
O1s (at%)	16.94	17.35	19.05	19.67

**Table 2:** Textural parameters of samples with different plasma treatments obtained from N_2_ adsorption analysis.

	AC	PR	P1	P2	P4
BET Surface Area (m^2^/g)	2791.30	2053.60	2019.00	2039.60	1928.00
Total Pore Volume (cm^3^/g)	1.61	1.17	1.16	1.17	1.12
Micropore Volume (cm^3^/g)	1.38	0.90	1.00	0.99	0.98
Micropore (%)	85.71	76.92	86.21	84.62	87.50

**Table 3:** Breakthrough parameters for HCl adsorption by PR, P1, P2, and P4 under identical fixed-bed conditions.

Sample Name	Flow Rate	Initial Concentration	Bed Height	Total Time	Breakthrough Time	Stoichiometric Breakthrough Time	Saturation Time	Effluent Volume	Total Amount Introduced	Total Removal	Adsorption Capacity	Length of MTZ	Bed Utilization
	Q	C_0_	Z	t_total_	t_0.05_	t_0.5_	t_0.95_	V_eff_	m_total_	R_total_	q_total_	L_MTZ_	f
	(mL/min)	(mg/L)	(cm)	(min)	(min)	(min)	(min)	(mL)	(mg)	(%)	(mg/g)	(cm)	(%)
PR	1000	50	2.5	91.3	20.3	47.4	85.4	91,300	4565.0	13.83	631.50	1.91	61.89
P1	1000	50	2.5	147.4	31.6	75.4	137.1	147,400	7370.0	13.50	995.26	1.92	61.52
P2	1000	50	2.5	99.7	28.7	59.4	95.5	99,700	4985.0	15.31	763.31	1.75	65.03
P4	1000	50	2.5	76.0	19.7	41.3	71.4	76,000	3800.0	14.20	539.51	1.81	63.80

## Data Availability

The original contributions presented in this study are included in the article. Further inquiries can be directed to the corresponding author.
